# Case Report: Identification of an HNF1B p.Arg527Gln mutation in a Maltese patient with atypical early onset diabetes and diabetic nephropathy

**DOI:** 10.1186/s12902-018-0257-z

**Published:** 2018-05-15

**Authors:** Nikolai Paul Pace, Johann Craus, Alex Felice, Josanne Vassallo

**Affiliations:** 1 Centre for Molecular Medicine and Biobanking, University of Malta, Msida, Malta; 2 Department of Obstetrics and Gynaecology, University of Malta, Msida, Malta; 3 Department of Medicine, University of Malta, Msida, Malta

**Keywords:** MODY 5, Diabetic nephropathy, Obesity, HNF1β, Atypical diabetes

## Abstract

**Background:**

The diagnosis of atypical non-autoimmune forms of diabetes mellitus, such as maturity onset diabetes of the young (MODY) presents several challenges, in view of the extensive clinical and genetic heterogeneity of the disease. In this report we describe a case of atypical non autoimmune diabetes associated with a damaging HNF1β mutation. This is distinguished by a number of uncharacteristic clinical features, including early-onset obesity, the absence of renal cysts and diabetic nephropathy. HNF1β-MODY (MODY5) is an uncommon form of monogenic diabetes that is often complicated by a wide array of congenital morphological anomalies of the urinary tract, including renal cysts. This report expands on the clinical phenotypes that have been described in the context of HNF1β mutations, and is relevant as only isolated cases of diabetic nephropathy in the setting of MODY5 have been reported.

**Case presentation:**

An obese Maltese female with non-autoimmune diabetes, microalbuminuria, glomerular hyperfiltration, fatty liver and no renal cysts was studied by whole exome sequencing to investigate potential genes responsible for the proband’s phenotype. A rare missense mutation at a highly conserved site in exon 8 of HNF1β was identified (c.1580G > A, NM_000458.3, p.Arg527Gln), with multiple in-silico predictions consistent with pathogenicity. This mutation has not been previously characterised. Additionally, several common susceptibility variants associated with early-onset obesity, polygenic type 2 diabetes and nephropathy were identified in the proband that could impose additional effects on the phenotype, its severity or its clinical course.

**Conclusion:**

This report highlights several atypical features in a proband with atypical diabetes associated with an HNF1β missense mutation. It also reinforces the concept that monogenic causes of diabetes could be significant contributors to disease burden in obese individuals with atypical diabetes.

## Background

The diagnosis of atypical non-autoimmune diabetes presents several challenges. These forms of diabetes are best exemplified by maturity onset diabetes of the young (MODY). MODY is a rare group of genetically heterogenous conditions characterised by beta cell dysfunction and defects in insulin secretion. Systematic screening of European and North American paediatric populations has identified a prevalence range of 1.2 to 4.2% [1–3]. Population-specific differences in the prevalence of MODY have also been reported [4, 5].

A wide array of phenotypic heterogeneity is observed in subjects with different mutations in the various genes implicated in the pathogenesis of monogenic diabetes, as this disease can mimic either type 1 or type 2 diabetes mellitus. Correctly making the diagnosis of MODY is essential in view of the therapeutic and prognostic implications. Furthermore, the autosomal dominant pattern of inheritance mandates genetic counselling and family follow-up. The increasing availability of whole exome or targeted capture followed by high throughput sequencing facilitates the molecular diagnosis of monogenic diabetes, particularly in cases where the clinical phenotype is atypical or complicated by clinical features that are not routinely associated with MODY. Increasingly, next generation sequencing followed by interpretation using publicly-available aggregate exome variant datasets offers an unrivalled scope for the discovery and annotation of novel variants.

Identifying patients with monogenic diabetes requires an index of clinical suspicion of the disease followed by molecular diagnosis to confirm the underlying genetic defect. Guidelines issued by the International Society for Paediatric and Adolescent Diabetes (ISPAD) are available to advise clinicians, and genetic diagnosis is available in many healthcare systems [6] . Despite these guidelines, studies have shown that genetic testing for MODY is under requested and clinical prediction models have been developed to aid the identification of likely candidates for molecular genetic testing [7, 8].

In this report, we describe a rare damaging missense mutation in HNF1β that was identified in a proband with early-onset atypical diabetes with no morphological renal tract anomalies. The proband had glomerular hyperfiltration and early stage diabetic nephropathy, which is unusual in the setting of MODY5. Because of the unusual features of nephropathy in the absence of renal cysts and obesity, we performed whole exome sequencing of the proband.

## Methods

### Patient

This study was approved by the ethics institutional review board of the University of Malta (IRB 71/2013). Written informed consent was obtained from the proband. The mother had demised and the proband’s brother and father were not available for genetic analysis at the time of the study.

### Sample preparation and whole exome sequencing

Genomic DNA was extracted from a peripheral blood sample taken from the proband using a QIAamp DNA extraction kit (Qiagen, Hilden, Germany), and checked for purity and integrity using agarose gel electrophoresis and UV spectrophotometry. Sample preparation and exon enrichment for next generation sequencing was performed using a SureSelectXT All Exon V5 kit (Agilent Technologies, Santa Clara, CA). 3 μg of DNA was processed according to manufacturer’s instructions. Paired end sequencing was carried out on an Illumina HiSeq 2500 sequencer (Illumina, Inc., San Diego, CA, USA).

### Exome sequencing alignment, variant calling and mutation detection

Image analysis was performed with the default parameters of Illumina RTA pipeline, and base calling was carried out using CASAVA. The sequence reads were mapped and aligned to the Human Reference Genome (UCSC hg19, NCBI build 37) using the Burrows-Wheeler transformation algorithm, and duplicated reads were removed using Picard [9, 10]. FastQC was used to check the quality of sequence data [11]. Calling of SNPs and InDels was done using GATK Unified Genotyper, which uses a Bayesian genotype likelihood model to report alleles and Phred-scaled confidence values [12]. Variants (SNVs and indels) were called with SAMTools, with reference to public databases including dbSNP and 1000Genomes and gnomAD [13]. Analysis was performed with preference to variants located in genes implicated in atypical non-autoimmune forms of diabetes and early-onset obesity. The prioritized candidate gene list was obtained by reviewing publications in PubMed and OMIM. Analysis focused on non-synonymous coding variants, frameshift indels, and variants affecting splice sites, as these are most likely to be pathogenic. Non-exonic and synonymous variants were excluded from further analysis. Missense variants were evaluated for functional impact using a variety of *in-silico* prediction tools including SIFT [14], Polyphen2 [15], MelaLR [16], MetaSVN [16], fathmm-MKL [17], DANN [18], CADD [19], MutationTaster [20], Mutation Assesser [21] and LRT [22].

### Sanger sequencing

Sanger sequencing using standard PCR amplification procedures was carried out to confirm the selected candidate variants of interest in the proband and in 300 unrelated controls of Maltese ethnicity. A 242 base pair region in exon 8 of HNF1β was amplified using the following forward primer (GGG CTC TGT ACC TGT GTC TT) and reverse primer (CCA TGG CCT TAT CAC ACC CT) with an annealing temperature of 54 degrees.

## Case presentation

### Clinical features

The proband is a 25 year old Caucasian female born from nonconsanguineous parents of Maltese ethnicity. She developed obesity in early childhood, with a body weight at the 97th centile at the age of 9 years. She was diagnosed with diabetes mellitus at age 11 following her presentation with osmotic symptoms of hyperglycaemia. No diabetic ketoacidosis at diagnosis was present, and both glutamic acid decarboxylase and islet cell antibodies were negative. She was initially treated by diet and lifestyle changes, and eventually started on metformin during childhood.

The proband became pregnant at age 21 years, and she delivered a healthy but macrosomic male infant by Caesarean section at 35 weeks of gestation weighing 5.18 kg. Her glycaemic control deteriorated significantly during pregnancy and was managed by combination treatment of isophane and soluble insulin. Significant weight gain also developed during pregnancy, with a BMI up to 37 kg/m^2^. Pre-proliferative diabetic retinopathy was also present in the proband.

Since pregnancy the proband developed persistent and significant microalbuminuria (urine microalbumin > 400 mg/L), leading to macroalbuminuria (albumin-creatinine ratio > 3000 mg/g) and glomerular hyperfiltration (eGFR >170mls/min/1.73m^2^) with normal creatinine levels. Urinalysis and urine microscopy showed no significant findings. Ultra-sonographic examination of the abdomen revealed normal size and echotexture in both kidneys, without any signs of obstructive uropathy, and normal cortical thickness and preservation of cortico-medullary differentiation. No evidence of autoimmune nephropathy or glomerulonephritis was present, with normal ANA, ANCA, C3, C4, rheumatoid factor IgM, uric acid, C-reactive protein and serum immunoglobulin levels. The proband also developed deranged liver function tests, with moderately elevated gamma glutamyl transferase and alanine transaminase levels. A viral hepatitis screen was negative, and hepatomegaly with no focal lesions and changes of a fatty liver were also evident on abdominal ultrasound.

As an adult, she is presently overweight (BMI 28 kg/m^2^) and glycaemic control is achieved by a combination of oral hypoglycaemic agents including metformin 1 g tds, gliclazide 80 mg tds and vildagliptin 50 mg daily. The proband however shows poor glycaemic control on combination oral treatment, with HbA1c values around 10%. Her fasting C-peptide concentration at the time of referral for genetic analysis was 1.4 ng/mL, indicating endogenous insulin production. Her HOMA-IR at the time was 2.8 (fasting insulin 5.7 mIU/L, fasting blood glucose 11.05 mmol/l). A repeat fasting C-peptide decreased to 1.1 ng/mL within 1 year. In addition, the proband was started on an angiotensin-converting enzyme inhibitor (perindopril, 8 mg/day) for reno-protection in view of the proteinuria and gradual increase in blood pressure that developed in the post pregnancy period. Her blood pressure control is generally well controlled on perindopril.

**Fig. 1:**
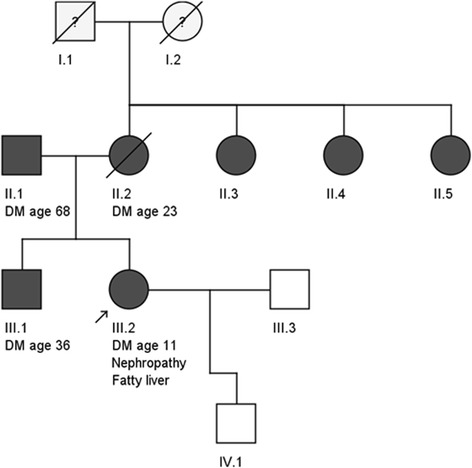
Pedigree of the affected family

The proband’s mother had developed diabetes at age 23, and was treated with oral hypoglycaemic agents and eventually insulin. The father developed type 2 diabetes aged 68 years, and the proband’s brother developed diabetes aged 36 years. In addition, three maternal aunts also had a history of diabetes mellitus. An overview of the pedigree is shown in Fig. 1.

In view of the above clinical picture, an initial diagnosis of early-onset type 2 diabetes complicated by fatty liver and diabetic nephropathy was made. The case was subsequently revised when the proband was offered and consented to genetic analysis for monogenic diabetes. In keeping with the strong family history of early-onset diabetes and the absence of ketoacidosis, the monogenic diabetes probability calculator showed a high probability of MODY (> 75.5% positive predictive value) [8]. Whole exome sequencing was the analytical method of choice, given the possibility of a primary genetic defect underlying the associated obesity, which is an unusual feature of monogenic diabetes.

### Mutation detection

A summary of the mapped sequencing data is shown in Table 1. WES yielded a total of 118,986 variants, of which 19,598 were classified as silent mutations, 16,869 as missense variants and 128 as nonsense mutations. Variants were filtered according to their frequency, location, functional consequences and clinical phenotype as outlined in the methods section.

**Table 1:** Whole exome sequencing detail of coverage and number of read

Number of reads in raw sequence	55,723,934
Number of reads after de-duplication	96.63%
Number of mapped reads	99.53%
Number of mapped reads to targeted regions of the genome	79.11%
Average depth	66.72%
Coverage 10×	98.67%
Coverage 20×	93.93%
Coverage 50×	58.10%

**Fig. 2:**
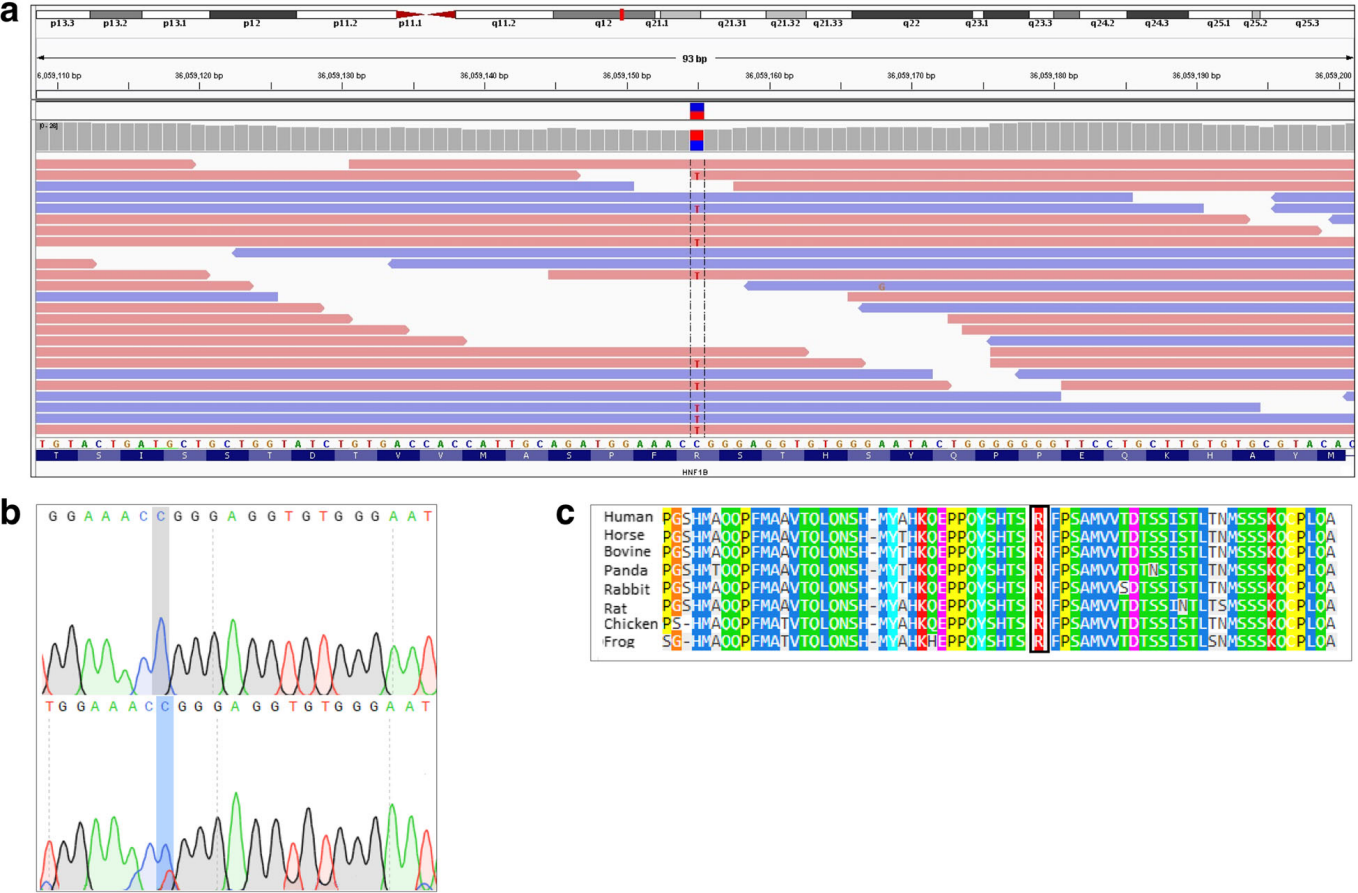
**a**: Excerpt of exome sequencing data visualised with Integrative Genomics viewer. The heterozygous C > T mutation at chromosome 17, position 36,059,155, in exon 8 of HNF1β is shown in the reverse strand. **b**: Sanger sequencing trace showing normal [top] and mutated [bottom] sequence. **c**: Multiple sequence alignment showing 66 amino acids surrounding the mutation position, indicated by the black box. The amino acid residue affected is highly conserved across multiple species

No nonsense, frameshift, in-frame indels and variants affecting splicing sites were detected in the proband in any of the common MODY genes. A heterozygous missense mutation with predicted damaging effects in HNF1β was identified in the proband - chromosome 17, position 36,059,155, c.1580G > A, NM_000458.3, p.Arg527Gln, exon 8. In-silico analysis of pathogenicity using various bioinformatic approaches are shown in Table 2. Clearly, multiple lines of computational evidence provide support for a deleterious effect of this mutation. Evolutionary conservation analysis also shows that the p.Arg527Gln mutation occurs at a highly-conserved position within the protein sequence, and that this residue is highly conserved across multiple species. The mutation was confirmed by Sanger sequencing in the proband (Fig. 2). In addition, the mutation was not detected by Sanger sequencing in 300 DNA samples of Maltese ethnicity. In the Genome Aggregation database (gnomAD), which reports summary data from large-scale exome and genome sequencing projects, the *HNF1β* p.Arg527Gln mutation has a very low frequency (3/246266, frequency = 0.00001218). This mutation lies in the C-terminal transactivation domain of the protein, and is predicted to be likely pathogenic based on the above criteria.

**Table 2:** In-silico predictors of pathogenicity and evolutionary conservation analysis for the HNF1βmutation described in the text

In-silico prediction of pathogenicity for HNF1β c.1580G > A (p.Arg527Gln)		
Tool	Prediction	Score
MutationTaster	Disease causing	
MutationAssessor	Medium	2.48
FATHMM-MKL	Damaging	0.9625
MetaSVM	Damaging	0.9934
MetalR	Damaging	0.9388
LRT	Deletrious	0
PolyPhen-2 - HumVar	Probably damaging	0.998
SIFT	Damaging	0.038
DAMN score	0.9996	
CADD scaled score		35
Evolutionary conservation analysis for HNF1β c.1580G > A (p.Arg527Gln)		
Genomic Evolutionary Rate Profiling (GERP)		5.63
PhyloP20way - mammalian		0.935
SiPhy29way - mammalian		0.9297

In addition to mutations in known genes implicated in monogenic diabetes, we also screened sequence data for the presence of genomic variants that have been associated in the literature with other phenotypes present in the proband, primarily obesity and nephropathy. A summary of the relevant findings is shown in Table 3.

**Table 3:** List of genomic variants and their respective minor allele frequency (1000 Genomes, TSI dataset) that have been identified in the proband

Chromosome	Position	dbSNP	Variant	Type	Gene	Effect	MAF	Clinical significance
chr3	10,331,457	rs696217	G/T	Missense	GHRL	p.Leu60Met	0.07	Risk factor for metabolic syndrome and childhood obesity
chr6	132,172,368	rs1044498	A/C	Missense	ENPP1	p.Lys173Gln	0.13	Susceptibility to Insulin resistance
chr4	6,292,915	rs10010131	A/G	Intronic	WFS1		0.36	Type 2 diabetes risk
chr6	149,721,690	rs237025	G/A	Missense	SUMO4	p.Val55Met	0.45	Type 2 diabetes risk, diabetic nephropathy
chr13	110,435,231	rs1805097	C/T	Missense	IRS2	p.Gly1057Asp	0.33	Type 2 diabetes risk
chr11	17,409,572	rs5219	T/C	Missense	KCNJ11	p.Lys23Glu	0.29	Type 2 diabetes risk
chr6	160,113,872	rs4880	A/G	Missense	SOD2	p.Val16Ala	0.47	Diabetic nephropathy
chr2	25,141,538	rs11676272	A/G	Missense	ADCY3	p.Ser107Pro	0.42	Childhood obesity

The variants in this table have been associated with obesity, type 2 diabetes and nephropathy in different studies

## Discussion

In the present report, whole exome sequencing revealed a rare missense mutation in HNF1β that shows high conservation scores and has in silico predictions in favour of pathogenicity. This mutation is located in the C-terminal transactivation domain of HNF1β, is absent from controls and is present at an extremely low frequency in the Exome Aggregation Consortium dataset. Furthermore, the proband’s uncharacteristic clinical phenotype and family history is suggestive of atypical or monogenic diabetes, although further evaluation of the mutation is required to provide a conclusive diagnosis of MODY5 in this case. Multiple prediction algorithms demonstrate a deleterious effect of this mutation on the gene. Missense mutations in HNF1β are commonly associated with both monogenic diabetes and kidney disease, and a number of in-vitro functional studies have demonstrated that missense mutations in HNF1β lead to impaired DNA-binding and reduced transactivation potential [23–25]. In view of these criteria, the mutation can be classified as likely pathogenic according to the established guidelines from the American College of Medical Genetics and Genomics and the Association for Molecular Pathology (ACMG/AMP) (criteria PP2, PP2, PP4, PM1, PM2) [26].

To date, around 230 mutations in HNF1β have been described [27]. This gene belongs to the homeodomain-containing family of transcription factors and is involved in the organogenesis of the kidneys, urinary tract, liver and pancreas. HNF1β functions as a homo- or hetero- dimer with a structurally related transcription factor HNF1α [28]. Mutations in HNF1α are responsible for the commonest type of monogenic diabetes (MODY3), characterised by progressive glucose intolerance due to an insulin secretory defect [7]. In contrast, mutations in HNF1β are associated with a wide array of clinical phenotypes that can include renal disease, and which are distinguished by the absence of clear genotype-phenotype associations. Primarily, heterozygous mutations in HNF1β cause a complex Renal cysts and Diabetes syndrome (RCAD) characterised by early onset diabetes (MODY5), liver dysfunction and pancreatic hypoplasia. The extent and severity of renal disease varies extensively in HNF1β mutations, ranging from congenital anomalies of the kidney and urinary tract (CAKUT), cystic kidneys to hyperuricaemia [29–31]. HNF1β mutations have been associated with isolated renal disease in the absence of diabetes and conversely, HNF1β deletions with young-onset diabetes but no kidney disease have also been described [32, 33]. Compared to other MODY genes, a high rate of de-novo mutations in HNF1β has been reported in the literature [29]. The variable expressivity of HNF1β mutations also extends to the type and age of onset of renal disease, which ranges from intrauterine life to middle age [34]. A recent multicentre retrospective cohort study of patients with HNF1β mutations showed that stage 3–4 chronic kidney disease was present in 44% of cases, and end-stage renal disease in 21% of cases [35].

The diabetic phenotype associated with HNF1β mutations is also equally heterogenous, with severity of glycaemia ranging from impaired glucose tolerance to diabetes requiring insulin therapy. The mean age of diagnosis of diabetes is 26 years, with a range of 10–61 years [36]. HNF1β mutations lead to beta cell dysfunction and reduced insulin secretion, which may be associated with pancreatic hypoplasia [37].

The exact cause of albuminuria and hyperfiltration in our proband is difficult to ascertain, as no evidence of renal cystic disease or of CAKUT was present on imaging. While renal cystic disease is a major manifestation of HNF1β mutations (65% of cases), considerable variation in renal phenotype exists. Similarly, studies have also shown that when renal biopsies are performed there is heterogeneity in the histological diagnosis [36]. Despite the presence of diabetes, the renal dysfunction found in carriers of HNF1β mutations is thought to be caused by renal development abnormalities rather than diabetic renal disease [38]. However, the proband also had microvascular complications of diabetes and poor glycaemic control, making associated diabetic nephropathy highly probable. A recent case report has described histologically-proven diabetic kidney disease (DKD) with glomerular sclerosis, severe diffuse mesangial cell proliferation, matrix expansion and arteriole hyalinosis in a 30 year old female with MODY 5 and macroalbuminuria caused by a missense mutation in exon 4 of HNF1β [39]. While glomerular hyperfiltration associated with early onset DM is a powerful risk factor for the development of progressive diabetic nephropathy, it is more likely attributed to glomerular DKD in our proband. Interestingly, another case report has described end-stage renal failure secondary to probable DKD in a patient with HNF1β mutation and RCAD syndrome [40].

We also examined exome data, focusing on susceptibility genes for early onset obesity, diabetic kidney disease and type 2 diabetes mellitus. Several common susceptibility variants were detected. Presumably, the additive effects of common risk variants contributes to the presentation, clinical course and progression of diabetes in the proband.

This report is significant for a number of reasons. Primarily, it expands on the mutational spectrum and wide array of clinical phenotypes associated with the HNF1β gene. In the era of personalised medicine, it is increasingly important to place the clinical phenotype into a gene-specific context that would enable clinicians to determine both immediate and future clinical implications to both the patient and family members. Secondly, it adds to the few clinical reports that highlight the presence of early-stage diabetic nephropathy with hyperfiltration due to probable glomerular DKD in the absence of renal cysts or renal morphological abnormalities in MODY5. Furthermore, it is important to emphasise that clinicians should maintain a high index of suspicion for monogenic diabetes in young onset non-autoimmune disease, and should consider referral for HNF1β mutation analysis in the presence of renal cysts with or without diabetes. The presence of obesity from a young age in the proband is also significant, in view of the fact that the initial diagnosis was of early-onset type 2 diabetes rather than monogenic diabetes. The report also reinforces the need for increased research on the molecular epidemiology of monogenic diabetes in the Maltese islands. As yet, the prevalence of MODY in Malta is not determined. It is likely to show significant differences from studies carried out in northern European populations, in part due to strong founder effects that have been reported on the island [41]. Of note, the same missense mutation in HNF1β has also been detected in an unrelated Maltese female referred for monogenic diabetes screening. The second case involves a 30 year old female with diabetes since age 13, a family history of paternally-transmitted early-onset diabetes, a BMI of 26.32 kg/m^2^, no history of diabetic ketoacidosis and treatment with long-acting insulin. Similarly, no renal cysts or structural renal tract anomalies were identified but investigation showed microalbuminuria from longstanding diabetes. The identification of the same missense mutation in two unrelated Maltese probands both with early-onset diabetes strongly suggests a possible genetic founder effect for this mutation. The authors are presently expanding genetic epidemiology studies to further investigate this.

## Conclusion

In conclusion, this report has described a rare missense mutation in exon 8 of HNF1β with multiple in-silico predictions consistent with pathogenicity. The proband had childhood onset atypical diabetes complicated by obesity and early stage diabetic nephropathy in the absence of renal cysts. The broad phenotypic variability of both diabetic and renal disease probably accounts for underdiagnoses of HNF1β mutations in clinical practice. The authors also emphasise that clinicians should be vigilant for the possibility of monogenic diabetes even in obese patients with a strong family history and a high probability on MODY risk calculators. This is particularly relevant in specific populations such as in the Maltese islands, where the population carries high prevalence rates for both obesity and diabetes, and the genetic epidemiology of the disease is as yet uncharacterised.

## Abbreviations

CAKUT: Congenital anomalies of the kidney and urinary tract
DKD: Diabetic kidney disease
HNF: Hepatocyte nuclear factor
MODY: Maturity onset diabetes of the young
RCAD: Renal cysts and diabetes
SNP: Single nucleotide polymorphism

## References

[1] Fendler W, Borowiec M, Baranowska-Jazwiecka A, Szadkowska A, Skala-Zamorowska E, Deja G (2012). Prevalence of monogenic diabetes amongst polish children after a nationwide genetic screening campaign. Diabetologia.

[2] Pihoker C, Gilliam LK, Ellard S, Dabelea D, Davis C, Dolan LM (2013). Prevalence, characteristics and clinical diagnosis of maturity onset diabetes of the young due to mutations in HNF1A, HNF4A, and Glucokinase: results from the SEARCH for diabetes in youth. J Clin Endocrinol Metab.

[3] Shepherd M, Shields B, Hammersley S, Hudson M, McDonald TJ, Colclough K (2016). Systematic population screening, using biomarkers and genetic testing, identifies 2.5% of the UK pediatric diabetes population with monogenic diabetes. Diabetes Care.

[4] Misra S, Shields B, Colclough K, Johnston DG, Oliver NS, Ellard S (2016). South Asian individuals with diabetes who are referred for MODY testing in the UK have a lower mutation pick-up rate than white European people. Diabetologia.

[5] Kanthimathi S, Jahnavi S, Balamurugan K, Ranjani H, Sonya J, Goswami S (2014). Glucokinase gene mutations (MODY 2) in Asian Indians. Diabetes Technol Ther.

[6] Rubio-Cabezas O, Hattersley AT, Njølstad PR, Mlynarski W, Ellard S, White N (2014). ISPAD clinical practice consensus guidelines 2014. The diagnosis and management of monogenic diabetes in children and adolescents. Pediatr Diabetes.

[7] Shields BM, Hicks S, Shepherd MH, Colclough K, Hattersley AT, Ellard S (2010). Maturity-onset diabetes of the young (MODY): how many cases are we missing?. Diabetologia.

[8] Shields BM, McDonald TJ, Ellard S, Campbell MJ, Hyde C, Hattersley AT (2012). The development and validation of a clinical prediction model to determine the probability of MODY in patients with young-onset diabetes. Diabetologia.

[9] Li H, Durbin R (2009). Fast and accurate short read alignment with burrows-wheeler transform. Bioinformatics.

[10] Picard. http://broadinstitute.github.io/picard. Accessed 7 May 2018.

[11] Cock PJA, Fields CJ, Goto N, Heuer ML, Rice PM (2010). The sanger FASTQ file format for sequences with quality scores, and the Solexa/Illumina FASTQ variants. Nucleic Acids Res.

[12] McKenna A, Hanna M, Banks E, Sivachenko A, Cibulskis K, Kernytsky A (2010). The genome analysis toolkit: a MapReduce framework for analyzing next-generation DNA sequencing data. Genome Res.

[13] Li H, Handsaker B, Wysoker A, Fennell T, Ruan J, Homer N (2009). The sequence alignment/map format and SAMtools. Bioinformatics.

[14] Kumar P, Henikoff S, Ng PC (2009). Predicting the effects of coding non-synonymous variants on protein function using the SIFT algorithm. Nat Protoc.

[15] Adzhubei IA, Schmidt S, Peshkin L, Ramensky VE, Gerasimova A, Bork P (2010). A method and server for predicting damaging missense mutations. Nat Methods.

[16] Dong C, Wei P, Jian X, Gibbs R, Boerwinkle E, Wang K (2015). Comparison and integration of deleteriousness prediction methods for nonsynonymous SNVs in whole exome sequencing studies. Hum Mol Genet.

[17] Shihab HA, Rogers MF, Gough J, Mort M, Cooper DN, Day INM (2015). An integrative approach to predicting the functional effects of non-coding and coding sequence variation. Bioinformatics.

[18] Quang D, Chen Y, Xie X (2015). DANN: a deep learning approach for annotating the pathogenicity of genetic variants. Bioinformatics.

[19] Kircher M, Witten DM, Jain P, O’Roak BJ, Cooper GM, Shendure J (2014). A general framework for estimating the relative pathogenicity of human genetic variants. Nat Genet.

[20] Schwarz JM, Cooper DN, Schuelke M, Seelow D (2014). MutationTaster2: mutation prediction for the deep-sequencing age. Nat Methods.

[21] Reva B, Antipin Y, Sander C (2007). Determinants of protein function revealed by combinatorial entropy optimization. Genome Biol.

[22] Chun S, Fay JC (2009). Identification of deleterious mutations within three human genomes. Genome Res.

[23] Kim EK, Lee JS, Cheong HI, Chung SS, Kwak SH, Park KS (2014). Identification and functional characterization of P159L mutation in HNF1B in a family with maturity-onset diabetes of the young 5 (MODY5). Genomics Inform.

[24] Barbacci E, Chalkiadaki A, Masdeu C, Haumaitre C, Lokmane L, Loirat C (2004). HNF1beta/TCF2 mutations impair transactivation potential through altered co-regulator recruitment. Hum Mol Genet.

[25] Raaijmakers A, Corveleyn A, Devriendt K, Tienoven V, Pieter T, Allegaert K (2015). Criteria for HNF1B analysis in patients with congenital abnormalities of kidney and urinary tract. Nephrol Dial Transplant.

[26] Richards S, Aziz N, Bale S, Bick D, Das S, Gastier-Foster J (2015). Standards and guidelines for the interpretation of sequence variants: a joint consensus recommendation of the American College of Medical Genetics and Genomics and the Association for Molecular Pathology. Genet med off J am Coll. Med Genet.

[27] Stenson PD, Mort M, Ball EV, Evans K, Hayden M, Heywood S (2017). The human gene mutation database: towards a comprehensive repository of inherited mutation data for medical research, genetic diagnosis and next-generation sequencing studies. Hum Genet.

[28] Mendel DB, Hansen LP, Graves MK, Conley PB, Crabtree GR (1991). HNF-1 alpha and HNF-1 beta (vHNF-1) share dimerization and homeo domains, but not activation domains, and form heterodimers in vitro. Genes Dev.

[29] Bellanné-Chantelot C, Chauveau D, Gautier J-F, Dubois-Laforgue D, Clauin S, Beaufils S (2004). Clinical spectrum associated with hepatocyte nuclear factor-1beta mutations. Ann Intern Med.

[30] Raile K, Klopocki E, Holder M, Wessel T, Galler A, Deiss D (2009). Expanded clinical spectrum in hepatocyte nuclear factor 1b-maturity-onset diabetes of the young. J Clin Endocrinol Metab.

[31] Bockenhauer D, Jaureguiberry G (2016). HNF1B-associated clinical phenotypes: the kidney and beyond. Pediatr Nephrol.

[32] Weber S, Moriniere V, Knüppel T, Charbit M, Dusek J, Ghiggeri GM (2006). Prevalence of mutations in renal developmental genes in children with renal hypodysplasia: results of the ESCAPE study. J Am Soc Nephrol.

[33] Edghill EL, Stals K, Oram RA, Shepherd MH, Hattersley AT, Ellard S (2013). HNF1B deletions in patients with young-onset diabetes but no known renal disease. Diabet Med J Br Diabet Assoc.

[34] Heidet L, Decramer S, Pawtowski A, Morinière V, Bandin F, Knebelmann B (2010). Spectrum of HNF1B mutations in a large cohort of patients who harbor renal diseases. Clin J Am Soc Nephrol.

[35] Dubois-Laforgue D, Cornu E, Saint-Martin C, Coste J, Bellanné-Chantelot C, Timsit J, et al. Diabetes, associated clinical Spectrum, long-term prognosis and genotype/phenotype correlations in 201 adult patients with hepatocyte nuclear factor 1 B (HNF1B) molecular defects. Diabetes Care. 2017;40:1436–43 10.2337/dc16-2462 28420700

[36] Bingham C, Hattersley AT (2004). Renal cysts and diabetes syndrome resulting from mutations in hepatocyte nuclear factor-1β. Nephrol Dial Transplant.

[37] Gautier J, Bellanne-chantelot C, Dubois-laforgue D, Wilhelm J, Boitard C, Clauin S, et al. Multi-organ damage in Mody5 related to mutations of the hepatocyte nuclear factor-1βgene. Diabetes. 2002;51 https://insights.ovid.com/diabetes/diab/2002/06/002/multi-organ-damage-mody5-related-mutations/1049/00003439. Accessed 9 May 2018.

[38] Magee GM, Bilous RW, Cardwell CR, Hunter SJ, Kee F, Fogarty DG (2009). Is hyperfiltration associated with the future risk of developing diabetic nephropathy? A meta-analysis. Diabetologia.

[39] Wang Y, Zhao Y, Zhang J, Yang Y, Liu F (2017). A case of a novel mutation in HNF1β-related maturity-onset diabetes of the young type 5 with diabetic kidney disease complication in a Chinese family. J Diabetes Complicat.

[40] Hegde P, Meldon A, Lamen L, Sharma D, Kalathil D (2017). An interesting unfolding of the diagnosis of hepatocyte nuclear factor-1 beta (HNF1β) monogenic diabetes. Pract Diabetes.

[41] Farrugia R, Scerri CA, Montalto SA, Parascandolo R, Neville BGR, Felice AE (2007). Molecular genetics of tetrahydrobiopterin (BH4) deficiency in the Maltese population. Mol Genet Metab.

